# Complete Genome of “*Candidatus* Phytoplasma rubi” RS, a Phytopathogenic Bacterium Associated with *Rubus* Stunt Disease

**DOI:** 10.1128/mra.01303-22

**Published:** 2023-04-26

**Authors:** Dominik Duckeck, Christina Zübert, Jan W. Böhm, Gaia Carminati, Bernd Schneider, Michael Kube

**Affiliations:** a Department of Integrative Infection Biology Crops-Livestock, University of Hohenheim, Stuttgart, Germany; b Department of Agricultural, Food, Environmental, and Animal Sciences, University of Udine, Udine, Italy; c Julius Kühn-Institut, Federal Research Centre for Cultivated Plants, Institute for Plant Protection in Fruit Crops and Viticulture, Dossenheim, Germany; Indiana University, Bloomington

## Abstract

The phytoplasma “*Candidatus* Phytoplasma rubi” is associated with *Rubus* stunt disease. The complete genome was determined by assembling Oxford Nanopore Technologies system-derived long reads, with short-read polishing with Illumina reads. The genome of strain RS, from Germany, is organized in one circular chromosome with a length of 762 kb.

## ANNOUNCEMENT

The phytoplasma “*Candidatus* Phytoplasma rubi”, a member of the 16SrV group (elm yellows group), is known as the causative agent of *Rubus* stunt disease in raspberry and blackberry cultivars (1). This phytoplasmosis is characterized by stunting, shoot proliferation, virescence, enlarged sepals, phyllody, flower proliferation, and fruit malformations, resulting in the decline of the host and subsequent economic losses (2, 3). Due to the lack of *in vitro* cultivation for phytoplasmas, genome information is critical for understanding the pathogen-host interaction and improving diagnostics. The “*Ca*. Phytoplasma rubi” strain RS was transmitted from a German Rubus caesius accession to the experimental host plant Catharanthus roseus by the parasitic plant Cuscuta europaea and is maintained in C. roseus by periodic grafting.

A sequencing library template was generated by cetyltrimethylammonium bromide (CTAB) extraction (4) with cuttings taken from the experimental host. The DNA concentration for generation of the shotgun libraries was determined using a Qubit fluorometer (Thermo Fisher Scientific, Darmstadt, Germany). Long-read sequencing was performed by using the rapid barcoding sequencing kit and a SpotON flow cell R9 on a MinION sequencer (Oxford Nanopore Technologies [ONT], Oxford, UK), which provided 441,170 reads with MinKNOW v22.10.10 (ONT) using the default settings except for the minimal read length, which was set to 1 kb. Short, paired-read sequencing (2 × 150 nucleotides) was provided by a commercial supplier, yielding 12,612,264 pairs (NGSelect DNA library on a NovaSeq 6000 system in paired-end 150-nucleotides read mode; Eurofins Genomics Germany GmbH, Constance, Germany). Reads underwent quality trimming in CLC Genomics Workbench v21.0.5 (Qiagen, Aarhus, Denmark) with default parameters. Read selection for improving the assembly was performed by BLASTN+ v2.9.0 comparison of all ONT reads against a custom-made database comprising “*Ca.* Phytoplasma” and C. roseus nucleotide sequence entries in GenBank, following the exclusion of C. roseus-assigned reads. The remaining ONT reads (*N*_50_ value of 9.337 kb) were assembled in Canu v1.9 (5) with the nanopore-raw option, resulting in a circular and gapless chromosomal sequence. Short-read polishing was performed in CLC Genomics Workbench v21.0.5 (Qiagen) with default parameters. Sequence overlap was identified by BLAST analysis (6) using the contig as query and subject. The chromosome start was set to the *dnaA* gene, and overlaps were removed with Artemis v18.2.0 (7). The chromosomal sequence was automatically annotated in RAST v2.0 (8), inspected by BlastKOALA v2.2 (9), and then manually curated in Artemis v18.2.0 (7). Annotation completeness was inspected by BUSCO v5.2.2 analysis (10), using 151 single-copy orthologs from the *Mollicutes* class as a reference. Assembly debris was checked in RAST and against the NCBI nonredundant protein database (https://blast.ncbi.nlm.nih.gov). In addition, taxonomical binning of the debris sequences was performed in MEGAN v6.18.2 (11). Unless otherwise stated, the default parameters were used for all software.

The circular chromosome of “*Ca*. Phytoplasma rubi” strain RS consisted of 762,251 bp, with a GC content of 23.13%, and contained 2 rRNA operons, 32 tRNAs, 685 protein-coding genes, and 202 pseudogenes. The coding of pseudogenes is largely associated with the mobilome elements of strain RS. BUSCO analysis revealed completeness of 93%, which is weighted as normal for 16SrV group phytoplasmas. No plasmids were identified.

### Data availability.

Raw reads have been submitted to the Sequence Read Archive (SRA) under accession numbers SRR22523853 and SRR22523854. The whole-genome shotgun project has been deposited in DDBJ/ENA/GenBank under BioProject accession number PRJNA906321. The version described in this paper is the first version, accession number CP114006.1.
